# Chicago Medical Society

**Published:** 1884-11

**Authors:** Liston H. Montgomery

**Affiliations:** Sec’y.


### SOCIETY PROCEEDINGS.

CHICAGO MEDICAL SOCIETY. *Stated Meeting, 14th July, 1884.*

*The President,* DR. D. A. K. STEELE, *in the chair.*

The meeting was well attended, the cases presented, and the papers read, of interest, but the crowded state of our columns compels us to abridge the secretary's report.

Dr. Franklin H. Martin read a paper on "A Successful Case of Abdominal Section for Pyo-Salpinx, Treated by Volkmann's Method." A brief report of this interesting and instructive case is appended.

Mrs. X, American, aged 29, weight 200 pounds, no children, but one three months miscarriage 4 years since, consulted me a year ago because of suspected pregnancy. She had many of the symptoms of pregnancy—pains in the breasts, morning sickness, enlargement of breasts and increased size of abdomen. She had flowed regularly, however, every month that pregnancy had been suspected. The ordinary digital examination was made at this time, but no decided diagnosis was made. I advised her to see me later, and in two months I made another examination, and with all the methods adopted was unable to satisfy myself as to the true condition. I at this time took her to Professor Byford, who, upon making an examination and hearing her history, which was at this time to the effect that she felt very decided fœtal movements together with the other characteristic symptoms, was inclined to pronounce it a case of pregnancy. The woman had continued to flow scantily every month. Five months later I saw the patient, and, with Dr. R. W. Bishop, gave her ether and made a very thorough examination. By bimanual manipulation, through both vagina and rectum, the small, undeveloped uterus was found crowded close up behind the symphysis pubis, and inseparable from a large firm-walled tumor, about the size of a five-months pregnancy, occupying Douglas' *cul de sac*, and extending upwards nearly to the umbilicus. This tumor occupied a position a little to the left of the median line. Owing to the corpulency of the patient and the great thickness of the abdominal walls, it was impossible to make a positive diagnosis as to the character of the tumor. The probe was introduced into the uterus, and it measured 2½ inches. The patient was again taken to Professor Byford, and after careful examination he pronounced it a fibroid of the uterus. He, however, with commendable conservativeness, advised me not to be in a hurry to operate. Although the woman was suffering greatly from the pressure of the tumor upon the bladder and rectum, and intense backache and other reflex manifestations, I waited two months for further developments. At the end of that time, after laying before the patient the true condition of affairs, she was ready and anxious for an operation.

The 15th of May, therefore, assisted by Doctors R. W. Bishop, L. L. McArthur, E. L. Hollister, L. T. Potter, Stanley Black and T. H. Swayne, Doctor E. J. Doerning also being present, I proceeded by abdominal section to remove the tumor. All necessary antiseptic precautions attended to, an incision was made, about eight inches in length, through the abdominal wall, reaching from near the umbilicus to the symphysis pubis. The omentum was found firmly adherent to the tumor,—the adhesions covering a space of three or four inches in diameter. The adhesions were ligated with cat-gut and carefully separated. It was now, after the omentum was separated, that I knew for the first time the true nature of the tumor. It was not a fibroid, but a large dilation of the fallopian tube of the left side. The walls were very thick and firm, and the tumor so generally adherent to the surrounding tissues that it would have been easy, even yet, to mistake it for a solid tumor. The sense of fluctuation was very indistinct, and only by introducing a needle of an aspirator was there no doubt left. The contents were pus. The tumor, upon further examination, was found to spring from the left horn of the uterus, and on the opposite side of its attachment to the uterus were the remains of the fimbriæ of the tube, and the ovary of the left side. The walls were of a muscular character, and very smooth. My intention was to remove this tumor entirely, clamping the small pedicle and attaching it in the inferior angle of the wound. But the tumor was found so universally adherent to the surrounding tissues that it was soon found impracticable to remove it with any reasonable hope of success. The intestines were firmly adherent upon almost its entire surface above, and the rectum was so closely adherent, that it seemed to be almost a part of the tumor. It was decided, therefore, after a short consultation, to resort to Volkmann's method of treating abscesses and similar tumors in other parts of the abdomen, viz: Stitching the walls of the tumor or abscess to the peritoneal edges of the abdominal wound and treating it, after union had taken place, as an external abscess. This was done in this case, the wound in the abdomen was closed as is usual, with the exception of the lower angle, where about three inches were left open. The tumor was stitched to the peritoneal edges of this open wound, so that when it was completed, about two inches in diameter of the upper surface of the tumor was exposed, and the peritoneum was closed. The whole wound was dressed antiseptically. The fourth day following the operation, the wound was exposed and the tumor was found firmly adherent to the abdominal wound. A free incision was now made into the tumor and a large double half inch drainage tube introduced to the bottom of the abscess. More than a quart of healthy pus was extracted, and the cavity was thoroughly washed out with a two per cent. solution of carbolic acid, by means of a fountain syringe, and that in turn (to prevent carbolic acid poisoning) was followed by a saturated solution of thymol. The abscess cavity was washed out thoroughly night and morning after this with a sol. of corrosive sublimate (1 to 3000) and the external wound dressed with corrosive sublimate gauze (1 to 2000). The patient continued to do well with this treatment until May 25th, the ten days following the operation, when the temperature suddenly rose up to 102° F., and the following day to $103\frac{3}{5}°$, the respirations were irregular and quick, and the pulse from 120 to 160.

At this time I called in Prof. Fenger in consultation. After a very careful examination of the patient, he decided that the lining of the abscess might possibly have taken on a change whereby it was absorbing pus and producing in that way the general constitutional disturbance. He recommended that the opening into the abscess be enlarged by blunt pressure, the inner surface of the walls be scraped with a dull curette and cauterized with a 20 per cent. solution of chloride of zinc. After the opening was enlarged, two fingers were introduced into the tumor and the abdominal cavity thoroughly searched by bimanual manipulation for any possible abscesses. A small abscess was found in the thick abdominal walls, and was opened and drained. The tumor was treated as recommended by Prof. Fenger, a larger drainage tube introduced. The temperature immediately returned to normal after this procedure, and has remained so. The tumor has gradually contracted and atrophied, the drainage tube has been shortened and made smaller, as was indicated from time to time as the cavity grew smaller, and at the present time is practically eradicated.

Dr. Martin, after a full discussion of the case by Drs. J. H. Etheridge, H. P. Newman, A. H. Burr, and L. L. McArthur, answered inquiries, and closed the debate.

Dr. S. W. Wetmore read a paper on "*The Rational Treatment of Fracture of the Clavicle*," illustrating it by adjusting his appliances to an assistant. The writer presented nothing new, but gave good descriptions of the methods devised and practiced by Drs. E. M. Moore, of Rochester, N. Y., and Lewis A. Sayre, of New York, respectively, making judicious comments on their relative advantages and disadvantages. The paper elicited remarks from Drs. R. W. Bishop, C. E. Webster, W. L. Axford, D. A. K. Steele, A. B. Strong, L. H. Montgomery, and W. H. Curtis.

The remainder of the evening was occupied by Dr. Jefferson Bettman in the reading of a carefully prepared paper on "*Rhinolithiasis*," embodying an interesting case which had occurred under the observation of the writer.

After some remarks by Dr. F. O. Stockton, the Society adjourned.

*Chicago Medical Society.*—Stated meeting, August 4, 1884. The President, D. A. K. Steele, M. D., in the chair. Dr. J. H. Etheridge read a graphic historical account of the treatment of cholera. Phosphorus was extensively employed before this century. Sixty years ago, calomel, opium, and venesection were the important therapeutic agents. James Johnson, in his classical work on *Tropical Diseases*, published in 1824, says,

"Start the bile, then support the powers of life." In the epidemics of 1826–27, the new remedies were carbonic acid, hydrocyanic acid, nitro-muriatic acid, chloride of gold, chloride of sodium, oxygen, arnica, buchu, purgatives, boiling hot water, cold water, counter-irritants, actual cautery, and chlorine. The epidemic of 1832–34 was a terrible one, and the list of therapeutic agents is long. Acids of all kinds, alkalies, albumen, bandaging, hot air and sand baths, vapor baths, belladonna, bile, tobacco, sulphate of copper, coffee, electricity, horseradish, transfusion, the injection into the bladder of medicated solutions, were employed in the treatment of the disease. An infusion of horseradish, calomel, and opium gained much reputation. Ice-water, intravenous injections of salines, iron, juniperberry oil, lead, lime-water, intravenous injections of milk, alcohol, turpentine, strychnia, the ligature of the extremities, croton oil, abdominal tourniquet, are also mentioned.

In 1848, anæsthetics, arsenic, camphor, capsicum, carbon disulphide, gum powder, hydrotherapy, *Perkins's tractors*, and ergot, were, in turn, remedial agents. In 1854, alcohol, chloride of calcium, eupatorium, garlic, potassium permanganate, quassia, sugar, *vaccination*, and *hard cider* were popular remedies. It was during this epidemic that B. W. Richardson, of London, advised the injection of water into the peritoneal cavity. About this time the nervous system began to receive attention from pathologists, and the nervines were introduced. Nitrite of amyl, calabar bean, coca, spinal ice-bags, hypodermic injection of water, and the dosimetric system received the attention of the profession.

In 1873, sulphurous acid, antimony, chlor-alum, were added to the list.

Dr. I. N. Danforth then discussed the Pathology of Cholera. He said that every organ of the body was more or less affected, but that pathologico-anatomical changes in the blood, intestinal walls, and kidneys were the three uniform lesions. The blood was thickened—sometimes to currant-jelly consistence—and contained microbes.

The intestinal villi were deprived of their epithelial covering, and Peyer's were enlarged. In the alvine discharges he had found, in 1873, epithelial cells, pus, blood, and microbes. Ziegler, Cornil and Ranvier, and Labarre (?) deny the existence of these epithelial cells in *ante-mortem* discharges. The arteries supplying the intestines were constricted, while the veins were greatly distended. The intestinal walls were, therefore, in a state of intense passive congestion. The kidneys presented the changes characteristic of acute fatty degeneration. The cortical glandular cells exhibited the fatty metamorphosis first. He agreed with Ziegler, Cornil and Ranvier, and Labarre (?) that the etiological relationship of Koch's microbe was not established. Dr. Danforth's remarks were well illustrated by drawings.

Dr. W. T. Belfield was called upon for information in relation to the Germ Theory of Cholera. He said that Dr. Robert Koch had found in the intestinal walls of cholera patients in India and Egypt a microbe recognizable by its size, form, and reaction to staining fluids. The microbe had not been found in any other tissue or organ. The constant association of this microbe with cholera was established. Its casual relationship was not proved. Koch had failed to produce cholera in dogs, rabbits, cats, and white rats, by the inoculation of the cultivated bacillus. He had seen the microbes in a rapidly multiplying condition in the intestinal walls. Until human animals were placed at the experimenter's disposal, Dr. Belfield was of the opinion that positive evidence as to the *rôle* played by the cholera bacillus would be lacking. In anthrax the etiological relationship of the anthrax bacillus had been clearly proved. In no other affection had the inoculation of cultivated bacilli produced the respective disease.

As regards the natural history of Koch's cholera microbe, it flourishes in alkaline solutions of a low degree of saturation, while acid liquids are not tolerated. It must be remembered that choleraic discharges are not usually alkaline.

Dr. John Bartlett, a member of the committee of three appointed, in 1873, to investigate the pathology of cholera, made some interesting remarks with reference to the *rôle* of the cholera bacillus. In 1874 he was engaged in experiments with "Saulsbury's ague fungus." He had observed a fungus—a cell —in a developing fluid, aggregate about itself a crystalline body, tangible and in quantities—bearing the relationship to the fungus that coral bears to the coral insect. This crystalline body in time put forth buds, which were of a green color. Presently, from these green buds, a clear protoplasmic mass was developed, which gave origin to cells, each one of which resembled the cell observed in the first place. May there not be some stage in the development of the cholera microbe when it is invisible, and at the same time functionally active?

Dr. Charles Gilman Smith made an able speech on the Prophylaxis of Cholera. He believed that the disease was highly contagious, and was accordingly surprised to hear a woman with the experience of Florence Nightingale, pronounce against the theory. The epidemic of 1854, which he had witnessed, furnished evidence sufficient to convince him that the disease was contagious, and that it was propagated by the decomposed dejecta of cholera patients. Without denying the possibility of its origin *de novo*, he knew of no recorded cases of such character. The disease, once developed, follows the line of travel. This theory was sustained by the fact that cholera was unknown in Australia. A thorough quarantine of ships and railroads should be instituted. Of the effectiveness of a thorough quarantine he had no doubt. In the last epidemic there was a cholera focus at Butterfield and Thirtieth streets, Chicago. The vigorous measures taken in removing the inhabitants and puri fying the premises had stamped out the disease.

As bearing upon the question of prophylaxis, a letter from Dr. John H. Rauch, Secretary of the State Board of Health, was read, of which the following extract is of interest:

The condition of the river, and especially of the South Fork, is a matter of the utmost importance to Chicago in the event of a cholera epidemic; and, indeed, at all times. Sixty thousand cubic feet per minute transferred from the river to the canal will keep the former clean and harmless; will prevent the possibility of pollution of the water supply at the crib; will affect sixty-six per cent. of the South Fork, and will so dilute the sewage as to make the canal unobjectionable to the communities along its banks. To secure this transfer requires two things:

1. That the pumps at Bridgeport be operated to their full capacity.

2. That the waters of the Desplaines River be prevented access to the South Fork through the Ogden Ditch.

If the Society can achieve this, it will render a most important service to Chicago, will help De Wolf in his arduous work, and earn the gratitude of one hundred and fifty miles of cities, towns, and hamlets along the canal and Illinois river.

Dr. N. S. Davis gave his experience in the epidemics of 1849, 1850, '52, '54, 1868, and 1873, on the Symptomatology and Treatment of Cholera.

Dr. Davis witnessed the epidemic of 1849 in New York city, and the others in Chicago. He did not believe in the germ-theory of the disease. In addition to an epidemic influence—whatever that might mean—there were three conditions necessary to cholera, viz., a high temperature, a soil in at least a damp condition, and the presence of decomposing organic matter. He was sceptical as to the efficacy of any quarantine.

There was but one affection with which cholera could be confounded—cholera morbus. The differential diagnosis was usually not a difficult matter. In cholera, except in the height of an epidemic, there is a premonitory, painless diarrhœa, lasting from a few hours to several days. Accompanying this diarrhœa is a feeling of lassitude, weakness, general *malaise*. In cholera morbus there is no premonitory diarrhœa, but vomiting and purging occur at once, with great intestinal pain. Following the premonitory diarrhœa, in cholera, the patient complains of dizziness, and prickling sensations over the skin surface, and particularly in the extremities. Soon the patient vomits the stomachic contents; then he discharges by the mouth and anus the characteristic rice-water evacuations of cholera. After the first up and down movement, paroxysms of vomiting and purging recur every ten or twenty minutes. The amount of this choleraic discharge is very variable, but may measure half a gallon at a time. If set aside in a glass vessel, flocculi settle at the bottom of the vessel, and the turbid, supernatant fluid becomes clearer. This fluid is composed of the serous elements of the blood, epithelial scales, and bacteria. In a fatal case, the patient now literally withers; symptoms of intense, acute anæmia supervene; the skin surface is alternately bathed with cold perspiration and shrivelled up. The eyeballs shrink back into their sockets; the finger-nails are blue, the lips pallid; the tip of the nose is cold, like a dog's; the breath is distinctly cool. Intense painful cramps are felt and seen in all voluntary muscles. The muscles of the calves, thighs, and upper extremities are seized first. The tongue is colorless, and the voice husky. The urine is entirely suppressed at an early stage of the disease.

In the height of an epidemic, the disease may run its course with frightful rapidity. In the epidemic of 1854, he had under his care a strong, healthy Scandinavian who, without any premonitory diarrhœa, was seized with serous vomiting and purging, and died three or four hours after the onset of the attack.

In cholera morbus, there is not this *ensemble* of the symptoms of acute anæmia, and the glandular secretions are not suppressed.

As regards treatment, he had few suggestions to make. In the premonitory stage, put the patient to bed at once—*when he is taken*—and treat as cholera morbus with the usual remedies when the patient commences to discharge by mouth and anus. Wait until the patient has become so exhausted by vomiting and purging that he can retch no longer, then put on the back of his tongue a powder containing one grain of calomel, one-sixth grain of morphia, and four or five grains of white sugar. He impressed upon his hearers the importance of doing this quickly, and said that it was folly to wait for stated periods to give medicines in such cases, for the chances were that a dose would be given first on the recurrence of the vomiting, and, therefore, no good would come. As an enema, he gave ten grains of acetate of lead and one-half grain of morphia, in two ounces of water, directly after a paroxysm of purging. Apply dry heat to the extremities ; sinapisms alternately to belly and back. After reaction has occurred, give dilute solution of table salt and small quantities of coffee and milk.

The cramps were best treated by firm compression. Rubbing was of less value. When vomiting ceased, he had been in the habit of giving, since 1849, small doses of the following mixture:

℞.—Carbolic acid..........................................gr. ¼

Tr. gelsemi.um..........................................♏ v

Paregoric..........................................f℥ ⅓

Water.................... ..................to f℥j.—M.

The thanks of the Society were tendered the venerable head of Mercy Hospital, and it was resolved to continue the discussion of the treatment of cholera at the meeting two weeks hence.

Regular meeting, August 18, 1884, D. A. K. Steele, M. D., President, and L. H. Montgomery, M. D., Secretary.

The first paper read was by Dr. P. C. Jensen, "On Cutaneous Therapeutics." After alluding to the histological and physiological relations of the skin and the internal secreting organs, including the mucous membranes, the writer discussed the question of cutaneous absorption, alluding to experiments in which limbs had been immersed in various solutions for weeks without any evidence of absorption. On the other hand, he quoted Bichat as authority for saying that when the cutaneous surface of a limb is immersed in putrid gases, absorption takes place, and the poison is subsequently eliminated by the bowels. He claimed that the rate and degree of absorption of any medicine depends largely upon its power of diffusion. Physiologically he regarded the skin as a "colloid septum, on one side of which lie the blood-vessels containing an alkaline fluid, while an acid is on the other side; a condition most favorable for osmosis." He quoted Waller to show that alkaloids dissolved in chloroform were readily absorbed from the cutaneous surface, while the same substances, dissolved in alcohol, were taken up very slowly or not at all.

He then gave systematically the objects and methods of local treatment in diseases of the skin, including a considerable number of formulæ, but which we must omit for want of space.

Dr. C. W. Earle read a short paper on the Management of the Summer Diseases of Children, of which we can give only the following conclusions:

1. The most frequent infantile disease in the city during the summer months is entero-colitis.

2. Excluding causes of infantile mortality largely beyond our control, improper feeding is one of the chief causes of the great number of deaths among this class.

3. Mothers should nurse their children. In lieu of this, a wet nurse should be procured. If this is impossible, a mixed diet is preferable, and, lastly, artificial diet must be resorted to.

4. Artificial foods containing considerable caseine are found to be a cause of indigestion and summer diseases.

5. In many cases cows' milk diluted with water does not seem to agree with children. Barley water, or rice water, as the diluent, seems to make a more physiological food.

6. Condensed milk seems to agree with a considerable number of children, but, in many cases, a sufficient quantity is not used to nourish a child. Used in proper quantities and diluted with rice or barley water, it is, without doubt, one of the best artificial foods.

7. Cream, mutton broth, and white of egg are valuable adjuvants to the dietary of infants.

8. Whatever the artificial food a child is having, the physician should examine frequently for evidences that it is a proper food as regards quality and quantity. The normal elevation of the fontanelles and increasing weight are among the conditions denoting a satisfactory and favorable nutrition.

The remainder of the evening was spent in discussing the subject suggested by the paper of Dr. Earle.

Stated meeting, 1st September, 1884, the President, Dr. D. A. K. Steele in the chair.

"Remarks on Aneurisms," by Dr. J. A. Robinson; "The Recent Treatment of Asiatic Cholera, as in vogue among European Surgeons of Late Years, in Southern India," by Dr. H. Martyn Scudder.

Dr. J. A. Robinson read a paper entitled, "Remarks on Aneurisms," and exhibited a specimen of an aneurism of the arch of the aorta, involving the aortic valves. The following abstract, which has been prepared by the secretary, we append. We omit the etiology, pathology, and diagnosis of aneurisms, and proceed to the general principles governing their treatment, which have been to prohibit the patient from taking much exercise, to secure, as nearly as possible, absolute rest, and to restrict the diet. Where the heart has been tumultuous, cardiac sedatives have been exhibited. Such symptoms as dyspnœa and pain have been met with appropriate palliatives. The first attempt at specific treatment for internal aneurisms was employed many years since by Albertini and Valsalva, and has been known as Valsalva's method. It consists in weakening the patient by repeated blood-lettings, and by gradually diminishing his meat and drink till only half a pound of pudding is taken morning and evening, with only a measured quantity of water; so that at last the patient is so exhausted he can not lift his hand from the bed in which he is ordered to lie from the commencement of the treatment. When this stage is reached, the amount of nutriment is increased until the patient's strength is restored. It is needless to say that this plan of treatment did not yield the beneficial results which were anticipated. In our day it would be regarded as barbarous, were we to try and enforce it to the extreme which Valsalva reached. A modification of this treatment, consisting of enforcing absolute rest in bed, and diminishing the food and drink, so as to diminish the quantity, but not the physiological quality, of the blood, has benefited a number of cases, if not entirely cured them. While some physicians have refused to employ the depleting treatment, they have resorted to measures fully as severe. Dr. Murchison and Mr. Moore, of England, have recommended, and in one case tried, the introduction of fine wire into the aneurismal sac, on the theory that the large amount of surface exposed to the circulating fluid would produce coagulation of the fibrin. In the case referred to, they introduced twenty-six yards of fine iron wire into the aneurismal sac, and it is needless to say that the treatment was unsuccessful, although they contend that the result obtained demonstrated that the principle was sound, and that further experiments were justifiable. A much less dangerous, and probly more efficient mode of treatment is by electrolysis. Pravaz was the first to use electrolysis for reducing external aneurisms, and Cinicelli and others have applied it to internal aneurisms, but with very different results. Its employment has been extended to thoracic and abdominal aneurisms, but, in eight cases of thoracic aneurisms, only one was benefited, the other attempts being unsuccessful. Only one case of abdominal aneurism was cured, and this patient died from rupture of the sac, on account of premature exertion. The results obtained from the use of the galvano-puncture have not justified us in expecting to hope for much from that method. Professor Langenbeck has published accounts of two cases which he claimed to cure by hypodermic injections of one-half to three grains of Bonjean's watery extract of ergot every three days. Balfour says he has tried this method frequently, but without any success, although he was positive that his ergot was active.

Pressure, as a mode of treatment, is wholly inapplicable to thoracic aneurisms, and rarely to abdominal. Dr. Murray records a case of abdominal aneurism in which pressure on the aorta for five hours, the patient being under chloroform, was successful.

We will now speak of the treatment of internal aneurisms by the administration of the iodide of potassium. It is conceded by Flint and Bramwell, and insisted on by Balfour, that the iodide of potassium is the only drug which offers any hope of cure, and that in every case it will relieve the distressing symptoms, if it does not effect a cure. Balfour says: "Of all the various modes of treating internal aneurism, there is not one hitherto mentioned which is not attended with considerable risk or danger, except Mr. Tufnell's plan of perfect rest, while the advantages to be derived from some of them are, to say the least, very problematical. But the treatment by the iodide of potassium, of which the author now writes, is perfectly safe and free from all risks, and it is equally certain to afford relief; at least no case has been yet found where relief was not attained, though, naturally enough, that relief is not always instantaneous, but requires the treatment to be continued for some time. It also relieves the pain and other symptoms of aneurism more rapidly and more effectually than any other treatment, apart, even from the powerful agency of the recumbent posture; and for the time it has been in use it has given greater and more permanent relief to a larger number of cases of aneurism than any other mode of treatment whatever. Indeed, the relief to the pain and other symptoms is so great and so speedily obtained, usually from the action of the drug alone, that it is often difficult to get the patient to submit to any restrictions; besides, it is not always necessary. The author has employed this method of treatment during the last eight years in a very considerable number of cases, with unvarying success, so far as the relief to symptoms is concerned, and with such favorable results as to retarding the farther progress of the case, and even, in some cases, promoting an apparent cure, as certainly to stamp this treatment as one of the most efficient hitherto propounded for the relief of this intractable complaint.

Balfour relates the history of twelve cases treated by this method, with the following results: The symptoms, such as pain, dyspnœa, etc., were relieved in every case; the physical signs of aneurism were diminished iu seven cases; pulsation of the tumor ceased in two cases, diminished in four, and was not apparent from the commencement in six. The aneurismal tumor disappeared in three cases and diminished in five; the bruit disappeared in two cases and diminished in two cases, but never existed in two cases. Five of the patients were so relieved that they could work, four were discharged at their own request, feeling well, one patient absconded, and the result of treatment in his case is not known. Five cases were termed cured, and seven were not cured, but relieved. One of the twelve cases referred to was an aneurism of the innominate artery, which was cured, and Balfour claims to have cured several cases of aneurism of this artery. One of the four patients discharged at their own request was under treatment three different times, being discharged twice at his own request, but died suddenly while under the third course of treatment, the autopsy revealing an aneurism of the aorta, which had ruptured into the lower lobe of the right lung. One of the twelve cases was diagnosticated as a weeping aneurism, implicating the origin of the left carotid, and communicating by a small opening with the left bronchus, the patient, on admission, expectorating arterial blood, but this soon ceased, and the patient was discharged cured. While the writer did not believe he was justified in being as enthusiastic in the praise of the iodide of potassium treatment as Balfour, he did believe he was justified in relating the following case, and giving the iodide of potassium the credit of prolonging the patient's life and making his life comparatively comfortable.

John H. C., aged 40, a blacksmith, was first seen in March, 1883. Had had attacks of inflammatory rheumatism several years ago. In February, 1883, was attacked with severe pains in the chest, in the præcordial region, and was treated by his physician for rheumatism for some weeks. Dr. J. P. Ross saw him and diagnosed aneurism of the aorta. At this time he had a good deal of dyspnœa, some hoarseness of the voice, and quite a little difficulty in swallowing solid food. When fatigued, the pains in his chest were excruciating. No tumor was perceptible, although there was pulsation in the upper sternal region and dullness upon percussion over the area of pulsation. Two months afterwards there was a swelling in the upper sternal region at the junction of the left first and second ribs, about the size of a silver dollar. A very slight bruit was heard. The voice was very husky, and the difficulty of deglutition had increased until now the patient could take no solid food whatever. He had emaciated, and was losing strength very rapidly. He was ordered to lie in bed continuously, and was given 15 grains of the iodide of potassium three times daily, gradually increasing the dose until signs of iodism appeared. It was truly remarkable how soon after this plan of treatment was inaugurated the patient expressed himself free from pain and the distressing symptoms from which he had suffered. He persevered this way until May, when he said he was so well and weary of the bed he would like to sit up. Leave was granted. A few weeks after, the writer was surprised to see him walk into his office. The patient complained of nothing. He was cautioned against such experiments, and he was told to return home and take better care of himself. His condition at this date was as follows: The continued pulsation of the tumor against the chest-wall had produced absorption of a large portion of the manubrium and an inch of the inner portion of the left first and second ribs. Consequently there could be felt the pulsations of the tumor through the chest-walls at a point where only soft tissues intervened. No bruit was discernible. On laryngoscopic examination we found complete paralysis of the left vocal cord. His voice was anserinous. During all these months he had been taking the iodide of potassium without any disturbing effects, until now, when he complained of symptoms of iodism. He was permitted to discontinue its use. From the date of this office visit he grew rapidly worse. He rapidly emaciated, dyspnœa and dysphagia increased, and finally he died of asthenia, July 19, 1884, about 17 months after he began treatment.

*Autopsy.*—On opening the thorax a large aneurismal tumor was seen behind the sternum, about five inches in diameter. Friable adhesions of the sac to the sternum were broken up when the sternum was removed. Absorption of a large portion of the sternum and the left first and second ribs had taken place. Heart and pericardium were normal. Adhesions between the aneurismal sac and the left lung. The left lung was pressed upward and backward into the left plural cavity, and collapsed. On opening the heart the aortic valves found roughened. Extending the incision into the arch of the aorta, find it dilated, and at the anterior of the arch, between the origin of the innominate and the left carotid arteries, an opening, oval in form, one inch by one inch and a half, into the sac of the aneurism. Through this opening the walls of the aorta were continuous, forming the wall of the aneurism. The tumor was firm, being composed of coagulated fibrine, and was then shown. Dr. Robison answered a number of informal inquiries relative to it, when Dr. John Bartlett cited briefly the history of a case of general chronic bronchitis and asthma, occurring in a man 70 years of age, who was supposed to be suffering from consumption for 50 years. Eight years ago, when the patient came under his observation, he ordered him to take eight grains of iodide of potassium three times a day. He has not omitted a day since to take the medicine, and he has steadily improved, his kidneys have performed their natural function, his appetite has not been impaired, and he has grown fatter and stronger. The case is an illustration how long a patient may take iodide of potassium continuously without injury to the mucous membrane of the stomach or injury to the kidneys.

These remarks were followed by the reading of a paper, entitled, "The Recent Treatment of Asiatic Cholera as in vogue among European Surgeons of late years in Southern India," by Dr. H. M. Scudder.

"I would not think of rising in the presence of so many of my seniors to give my views on the important subject of the treatment of cholera, if it had not been my fortune (or misfortune many might term it) to pass through four epidemics during my practice in India, where I labored for nine (9) years as a purely medical missionary. In the course of these epidemics I was called upon to treat a large number of cases, as I was at the head of a large hospital, supported by the English Government, and was the only European physician in a large town of nearly fifty thousand (50,000) inhabitants. During the famine of 1877 and 1878 I was placed in medical charge of a large relief camp, which frequently contained over five thousand (5,000) persons, and where I had two fairly trained native doctors to assist me. In 1878 the cholera broke out in this relief camp and raged as an epidemic for several months; the number of deaths in the camp often exceeding fifty (50) per diem. As it is now almost time to adjourn, I shall only touch upon a few points, outlining the form of treatment pursued of late years by many of the surgeons in India. For purposes of treatment I would divide the course of the disease into the following stages: A prodromic stage, a stage of diarrhœa, a stage of invasion, a stage of collapse, and a stage of reaction.

"In the prodromic stage, manifested by malaise, chilliness, depression of spirits, nausea, and abdominal discomfort, give ten or fifteen-drop doses of spirits of camphor, in dessert-spoonfuls of hot brandy, but be very careful not to give any considerable quantity of stimulants. As soon as the stage of diarrhœa begins, the administration of some preparation of opium, together with aromatics of camphor and a little chloroform is called for. Two parts of chloroform to one of spirits of camphor, is a very good combination; thirty drops for a dose. To be repeated as required. An equally serviceable one consists of equal parts of spirits chloroform, spirits camphor, laudanum, aromatic tincture of rhubarb and tincture of ginger. Teaspoonful doses every hour or two, until four or five doses have been given. In alternation, also, with either of these, a mixture of aromatic sulphuric acid and tincture of opium may be given with benefit. As soon as frequent vomiting commences and the stage of invasion becomes established, these combinations containing opium had better be discontinued, and in their stead, simple mixtures of aromatics, chloroform and hydrocyanic acid be administered by the mouth, to check vomiting, and enemas of acetate of lead given per rectum, while at the same time we resort to the administration of morphine, or morphine combined with chloral hydrate, by hypodermic injection.

"The patient should be kept perfectly quiet, covered with heated blankets, and dry heat applied to the surface of the body, especially to the extremities, by means of hot bottles, heated flat-irons, etc. The hypodermic use of morphine and chloral is, of course, contra-indicated when the stage of collapse has become well developed. During the stage of collapse it is essential that the patient be kept in the horizontal position. No violent rubbing should be allowed, but I have found it beneficial to gently rub the limbs and extremities with hot oil. *Very small* quantities of alcoholic stimulants are useful in this stage, but they should be given with great caution, lest vomiting should be provoked. Stimulant enemas may also be given.

"When the stomach is intolerant it is often better to inject small quantities hypodermically; experience teaches us, however, that anything like the free use of alcoholic stimulants is uncalled for and exceedingly harmful. I have sometimes found small doses of both atropia and strychnia, administered by hypodermic injection, apparently effectual in bringing about reaction. I have tried the intravenous administration of milk and salines in twenty-four cases, with only four recoveries. The reaction thus induced is generally not permanent, so I have been induced to abandon this method. I believe the impregnation of the air of the room with sulphurous acid by the burning of sulphur is a useful adjuvant in the treatment of cholera, and think it decreases the liability of the disease being propagated or contracted by the attendants. I see it is time for me to close, and will, therefore, not go into treatment of the stage of reaction and the further after treatment of the patient." In answer to a question, Dr. Scudder replied, "I consider that cholera is an infectious disease, and also somewhat contagious, though not highly contagious or readily communicable by personal association with the sick, as is the case with small pox or measles. The noxious power of the cholera germ, or virus (whether it is Dr. Koch's microbe or something else), seems to be more powerfully exerted some time after it has escaped from the body of the patient than when it is freshly passed. Careful observation and experience, the world over, seems to establish this fact. Whether the disease is contagious or highly contagious, however, seems still to be the vexed question, and remains yet to be decided. My personal experience in four epidemics and the careful study of other Indian epidemics has led me to believe that the attendants and those who come into frequent and close contact with cholera patients are somewhat more apt to contract the disease than those who do not."

Dr. Scudder's paper elicited a lively discussion, in which Dr. John Bartlett, J. H. Etheridge, R. E. Starkweather, D. O'Shea, G. Newkirk, G. C. Paoli participated.

Dr. Scudder, in closing the debate, said, first, as to the per cent. of recoveries where the epidemic was severe, it was 20 or 30 per cent., or the percentage of deaths ranged from 80 to 90 per cent. where the epidemic was very severe. If the cases were seen early the fatality ranged from 40 to 50 per cent. In the sheds where cases were treated by fumes of sulphurous gas in the premises, nearly half the cases might be saved, whereas, in one shed in the relief camp, containing 20 cases, where exactly similar conditions were present, where fumigation was omitted, 80 per cent. died.

In India the natives would not drink well-water. Their religion was the cause of frequent visitations of cholera, for it taught them to drink only that which descended from heaven. The result was, they drank surface water, or that which was stored in tanks near the temples of Lateran, which they consider as sacred. In contrast to the natives of India were the Chinese and Japanese, because they drank well-water, and used the garbage and human offal to fructify the fields, and they were comparatively free from cholera. The Indians would not remove excrement and offal, neither did they cover it up, and when the rain descended, which amounted to 8 or 10 inches sometimes a night, the water became polluted. The speaker did not see that any benefits would be derived by people running away from a district in which cholera was epidemic. General panic predisposes to the disease, and fright had much to do with its fatality. In India the poisoning of persons afflicted with cholera by arsenic was frequent, and is largely undetected by the police. To warn the community, as one gentleman has alluded to, is an extensive topic. If the early known methods of checking it were resorted to, cholera would not become epidemic. The Indians are greatly prejudiced against European medicines, but, singularly enough, not so with European surgery, presumably because there are no native surgeons in India.

No European would travel without a bottle of camphor, and the English are always provided with a flask of brandy. The Europeans are well up in the use of preventive measures, and they would be sure, also, to be provided with chlorodyne, which is no longer a secret remedy.

LISTON H. MONTGOMERY, M.D., *Sec'y.*

